# Layout of anatomical structures and blood vessels based on the foundational model of anatomy

**DOI:** 10.1515/jib-2024-0023

**Published:** 2024-07-12

**Authors:** Niklas Gröne, Benjamin Grüneisen, Karsten Klein, Bernard de Bono, Tobias Czauderna, Falk Schreiber

**Affiliations:** Department of Computer and Information Science, 570542 University of Konstanz, Konstanz, Germany; Independent Scholar, Halle (Saale), Germany; Whitby et al, LLC, Indianapolis, IN, USA; Auckland Bioengineering Institute, University of Auckland, Auckland, New Zealand; Faculty of Applied Computer Sciences & Biosciences, University of Applied Sciences Mittweida, Mittweida, Germany; Faculty of Information Technology, Monash University, Clayton, Australia

**Keywords:** blood vessel layout, foundational model of anatomy, anatomical structures, visualisation

## Abstract

We present a method for the layout of anatomical structures and blood vessels based on information from the Foundational Model of Anatomy (FMA). Our approach integrates a novel vascular layout into the hierarchical treemap representation of anatomy as used in ApiNATOMY. Our method aims to improve the comprehension of complex anatomical and vascular data by providing readable visual representations. The effectiveness of our method is demonstrated through a prototype developed in VANTED, showing potential for application in research, education, and clinical settings.

## 1 Introduction

The Foundational Model of Anatomy (FMA) [1, 2] is an evolving ontology of anatomical structures in humans. It represents a coherent body of knowledge about human anatomy, primarily aiming towards support of automated reasoning about human anatomy and thereby bridging the gap between computational resources and the continuous expansion of knowledge in biomedical sciences. The FMA ontology is hierarchically structured and includes various levels and types of anatomical entities and their relationships. It covers different aspects of anatomical structure, including anatomical entities (such as organs, tissues, and cells), spatial relationships (how entities are spatially related to each other, such as “is part of” or “is adjacent to”), and functional relationships (functional aspects like connections and dependencies between structures). As for the branches, the FMA is divided into major categories, each representing different aspects of human anatomy. It provides a comprehensive map of human anatomy, making it one of the most detailed resources available for understanding the complexities of the human body at various biological levels. The FMA has become a fundamental resource in bioinformatics, providing a scaffold for many applications including research, education, and clinical informatics. It is also used for integration and retrieval of biomedical information across various databases and systems.

**Figure 1:**
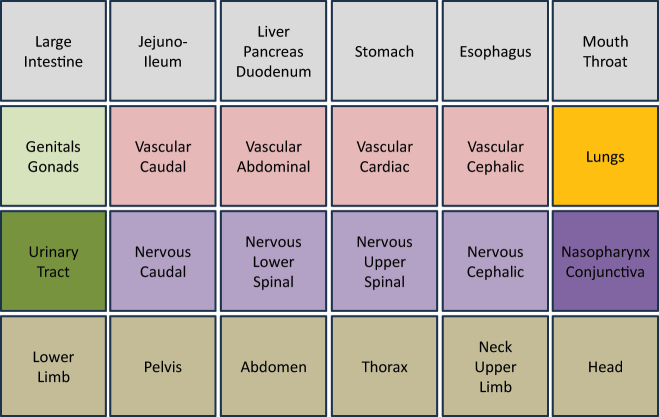
Initial view of an ApiNATOMY-inspired body layout showing the top-level 24 tile body anatomy plan, redrawn from [3]. A lateral cross-section of a human body (organs and regions are colour-coded) is abstracted to a 2D map with the 24 tiles representing body areas and organ systems.

**Figure 2:**
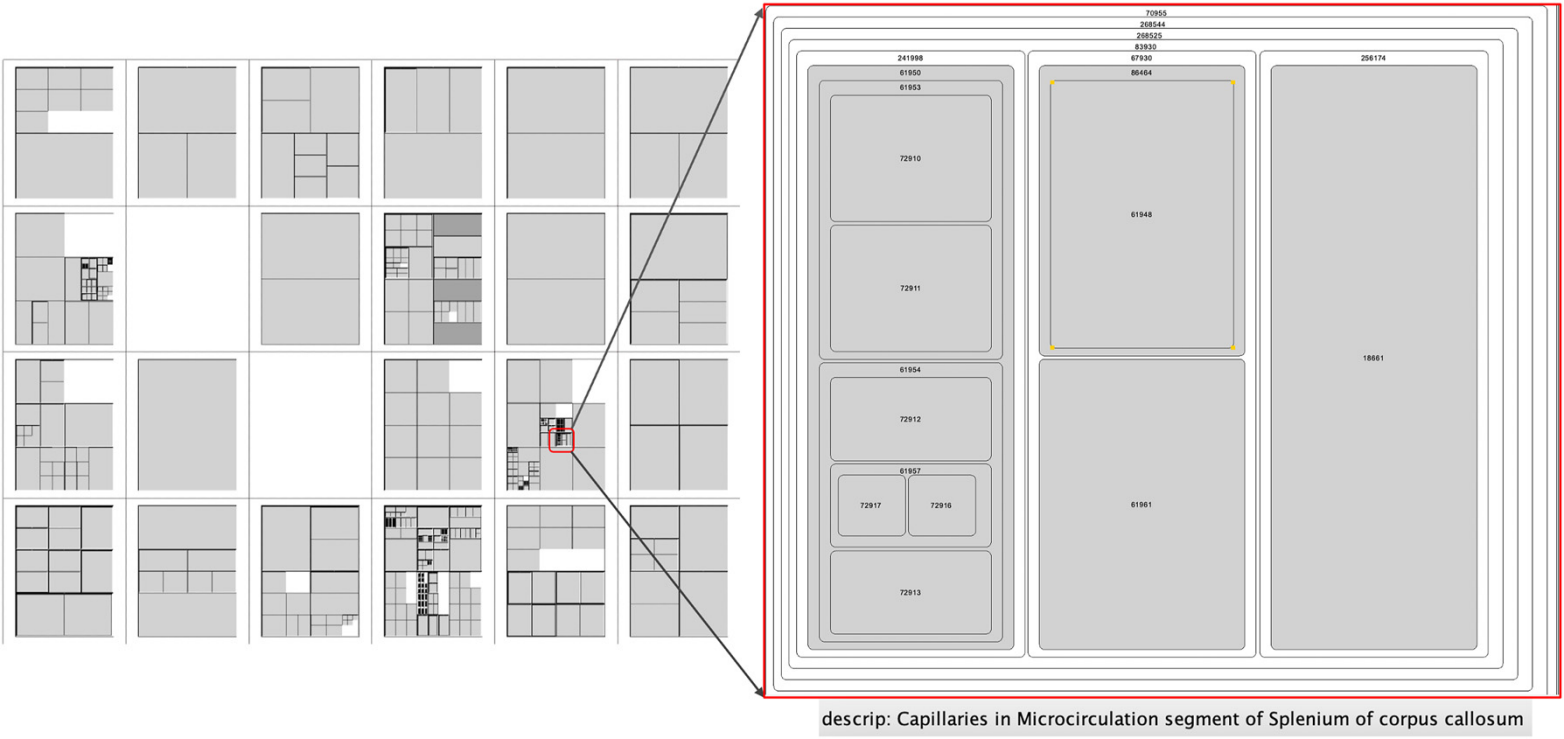
Example of treemap layout in the 24-tile structure, with an enlargement of one tile.

One of those applications is ApiNATOMY [3], an initiative designed to bridge the gap between a physiology expert’s knowledge and a broad spectrum of data pertinent to physiology, utilising an intuitive graphical interface that facilitates the management of semantic metadata and ontologies related to physiology. The web-based ApiNATOMY platform utilises the FMA and enables physiology experts to explore schematic, circuitboard-like visualisations of body parts and their cardiovascular and neural linkages at multiple scales [4]. These visualisations are enhanced with graphical depictions of organs, neurons, gene products, and mathematically modelled processes that are semantically annotated with relevant knowledge. ApiNATOMY offers a treemap-like layout [5] of the FMA structure that emphasises the hierarchical structure of organs and tissues. The human body’s physiology is divided into 24 anatomical compartments, so-called tiles, whereby the positioning of the compartments is carried out according to the model of a lying person, see Figure 1. The hierarchical information of the FMA is then shown inside the tiles as treemaps, see Figure 2, details are described in [3]. A blood vessel system can be integrated into this representation in a following step, blood vessels connect the parts of the FMA compartments. The arteries and veins are superimposed on the treemap representation, for example as differently coloured blue and red edges. However, the arrangement of these blood vessels (their layout) has not been adequately addressed so far. The resulting challenge is not only relevant for ApiNATOMY but for all cases of combining a superimposed hierarchy with a space-filling approach. A solution that represents an easily readable layout can significantly improve the understanding of the overall diagram with the aim that the user receives a clear, hierarchical visualisation of the physiological data and a representation of the connectivity of the individual anatomic compartments via blood vessels. A method for such a blood vessel layout will be presented in this paper.

## 2 Related work

### 2.1 The foundational model of anatomy

The Foundational Model of Anatomy (FMA) ontology [1, 2] is an open-source knowledge resource for biomedical informatics serving as a domain ontology that represents the structural organisation of the entire human body. The FMA distinguishes itself from traditional anatomical sources by providing a detailed, symbolic representation of anatomical entities and their relationships, supporting various applications in biomedical informatics. With over 75,000 classes and extensive relational data, the FMA facilitates cross-disciplinary integration and standardisation in anatomy representation. It structures anatomical information across different levels of abstraction into four main components: (1) Anatomy Taxonomy (AT), (2) Anatomical Structural Abstraction (ASA), (3) Anatomical Transformation Abstraction (ATA), and (4) Metaknowledge (Mk). This classification facilitates the detailed representation and understanding of the human body’s anatomy for various applications in medical and other research fields.

### 2.2 Treemap layout

For the visualisation pipeline (see Section 4 and Figure 5), we need to display the hierarchical relationship of the underlying anatomical system in a way that is easy to read and fast to interpret. For this a treemap layout has been proven useful [3, 6]. A treemap layout is a layout algorithm that recursively maps nodes of a tree into a, in our case, two-dimensional (2D) space, with the additional restriction to display the parent-child relationships of the underlying graph by placing a child node inside the layout area of its parent node [5]. A second condition that has to be fulfilled for a treemap layout is that the layout elements of any sibling nodes, i.e., nodes with the same parent node, must not overlap.

Over the years, several treemap layout algorithms have been created with different goals, such as achieving a circular layout [7], focusing on different attributes like weight, or preserving neighbourhoods. For the visualisation pipeline used here, we focus on the subclass of treemap layout algorithms that allocate nodes to a rectangular reference space. One of the first algorithms, the *Slice & Dice* algorithm introduced by Johnson and Shneiderman [8] follows the approach of recursively splitting the reference space along one axis, resulting in horizontal or vertical slices. Due to the limitation of only being able to split the reference space along one axis, this treemap layout approach may contain numerous thin and long slices, leading to a vast number of *splitting* treemap layout algorithms proposed [5]. For example, the *Squarrified* algorithm [9], extending the *Interactive Dynamic Map* algorithm [10], was introduced, focusing on achieving an aspect ratio of the to-be-created rectangular subdivision of one, or the *Golden Rectangle* layout approaching the golden ratio [11].

In addressing the trade-off between order preservation and aspect ratio, we note that conventional algorithms often compromise visual quality by incorporating various parameters not needed for our specific use case. Consequently, we propose a simplified treemap algorithm, the *Grid-Layout*, primarily focusing on enhancing visual quality. This approach deliberately overlooks many common parameters considered in existing algorithms, simplifying the overall approach and enabling the optimisation of the visualisation. A brief overview of this algorithm is outlined in Section 4, and a visualisation is shown in Figure 2.

### 2.3 Network layout

Network layout algorithms provide methods to display complex network data, including biological networks, in a comprehensible manner [12–14]. These algorithms aim to represent networks – comprising nodes and edges – in a way that conveys the structure of the data and allows to analyse it with low effort. Common approaches include force-directed algorithms [15, 16], which simulate physical forces among nodes and edges to position closely related nodes together while dispersing unrelated ones. Another approach is the hierarchical layout [17], which arranges nodes into levels based on their connectivity, often used in organisational charts or tree structures. An alternative method is circular layout [18], ideal for emphasising cyclic structures in data. Orthogonal layouts [19] arrange nodes and edges in a grid format, ensuring that all connections between nodes are represented by horizontal or vertical line segments. Each algorithm has its strengths and restrictions. Recent advancements have introduced hybrid algorithms that combine features of multiple techniques to handle specific data characteristics and layout methods with constraints [20–23], improving the visual outcomes of network visualisations and supporting important aspects such as mental-map preserving layout [24, 25].

### 2.4 Edge routing

Edge routing is the part in network visualisation that determines the paths between nodes for connection representation to produce a clear depiction in which edges and paths are easy to follow and the network structure is easily perceived. Typical goals for edge routing are to minimise edge crossings, edge length, and overlap with nodes, thereby reducing visual clutter and improving readability [26]. Effective edge routing is crucial when dealing with complex graphs where a straight-line connection between nodes would result in an unreadable and potentially confusing drawing.

There are several strategies and styles for edge routing, including orthogonal routing [27], where edges follow right-angle paths; spline routing, where edges are drawn using curved lines such as Bézier curves [28]; and polyline routing [29], where edges are piecewise linear but not necessarily orthogonal. The choice of strategy and style often depends on the application and the specific requirements for the visualisation, such as whether the graph is directed or undirected, the density of the graph, and the need for highlighting certain pathways or structures within the graph.

Advanced edge routing algorithms also take into account aesthetic criteria and constraints, such as minimising the number of bends in an edge to reduce visual complexity or using edge bundling [30, 31] to group similar edges together, which can help in revealing higher-level structures within the graph. Edge routing can be particularly challenging in dynamic graphs where the nodes and edges change over time, requiring the routing algorithm to adapt while maintaining a stable and mental-map preserving layout.

## 3 Data

The underlying base data was retrieved from the Foundational Model of Anatomy (FMA) ontology [1, 2]. The following steps can be used to extract the data from the FMA and format it for the visualisation pipeline.

**Figure 3:**
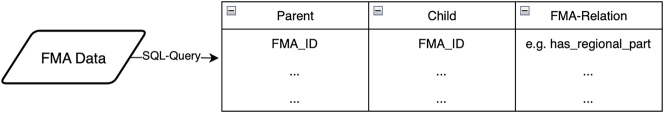
Schematic of the anatomical data derived for the tree map layout.

**Figure 4:**
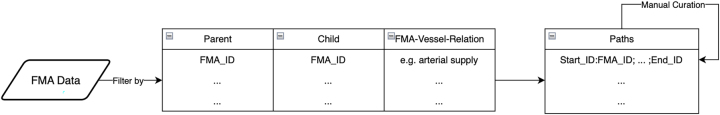
Schematic of the vascular data derived for the vascular layout.

– First, a SQL query is used to pull the complete raw data from the FMA ontology. This query should target entities based on four hierarchical principles such as *Constitutional part*, *Regional part*, *Direct Subclass*, and *Direct Instance of.* It should be noted that, due to its complexity, more than a single hierarchical principle is required to represent the complete structure of the body. For example, the hand is divided into fingers, a thumb, and a palm, each with further subdivisions into bones and joints. Similarly, the abdominal region is described through close yet distinct organs like the intestine, liver, spleen, and pancreas, categorised under *R. part.* These categories should include specific entity names or identifiers related to these hierarchical principles, ensuring the precision of the data extraction process.
– Second, the resulting SQL file is then filtered into a table (see Figure 3) containing entities and their respective relational connection like the following:
<FMA-Parent_Id> <FMA-Child_Id> <Relational Connection>
  It should be noted that curation and verification steps on the extracted data are required to ensure a feasible data set, which is represented by a tree. Retrieving a body hierarchy directly from the FMA database may result in loops, leading to a failed layout.
– Third, the information for the blood vessel system is retrieved. To retrieve the base data of the vascular system, understanding the hierarchical principles used in the FMA to represent vascular structures is essential. Fundamental principles include *arterial supply* for arteries, which is documented for over 17,594 vessels, and *venous drainage* paths like *venous drainage of*, *receives drainage from*, and *drains into.* Unlike arterial systems, a comprehensive equivalent for venous systems is less clearly defined in the FMA. The data can be extracted from the FMA using specific search criteria focusing on the vascular system, mainly targeting the relational attributes of arterial and venous systems (see Figure 4, left). Since no single root node exists for the vascular system, the heart often acts as a central reference point for vascular data extraction. The extracted vascular paths, composed of FMA-IDs of corresponding microcirculations,1Microcirculation is the vascular network of the systemic circulation consisting of microvessels with diameters < 20 µm [32]. can then be saved in a separate file.
– Fourth, given the complexity of the vascular data and incomplete nature in the FMA, the detailed mapping requires manual curation and verification (see Figure 4, right). This step is crucial for accurately representing transitions between different vascular segments and ensuring the entire graph reflects the anatomical data as closely as possible.

## 4 Methods

**Figure 5:**
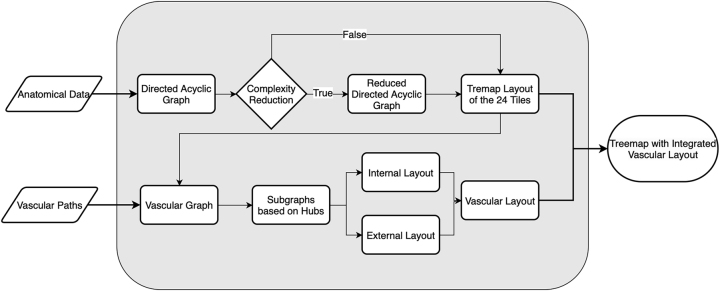
Visualisation pipeline.

The visualisation pipeline (see Figure 5) comprises two distinct steps. The first step involves generating a treemap representation of the human body’s hierarchy based on the body in a lying position, organising the anatomical data into 24 distinct, nested segments called *tiles* as described in [3]. A tile, therefore, refers to a functional body part whose task, tissue type, or physiological function is uniform within the tile. The second step of the pipeline overlays a vascular system connecting to microcirculations of the treemap layout.

### 4.1 Treemap layout

For the first step of the visualisation pipeline, a directed acyclic graph (DAG) is divided into 24 sub-trees representing individual tiles based on the retrieved anatomical base data set. Each tile can be different in size or even be empty and is assigned to specific sections of the treemap. A global scaling factor regulates the size of each tile.

To enable a mechanism for reducing complexity and thereby customise the level of detail for specific applications, the microcirculations, which serve as connection points for the vascular layout mentioned later, can be limited to a predefined set. As a result, vascular treemap paths are created solely based on the selected set of microcirculations. Without such a selection, the treemap incorporates all microcirculations, treating them as potential connection points for the vascular layout.

Another way of simplifying the complexity of the system is to limit the level of detail shown within a tile in the treemap layout. This is done by filtering the nodes to be displayed based on their distance to the root node. In the context of the FMA, a global maximum distance can be set ranging from 1 to 16, adjusting the visibility of the nodes so that only those within the specified distance are displayed in the treemap layout.

Lastly, a tree mapping algorithm, chosen from three options (Grid-Layout, Slice&Dice, or Squarrified), generates the treemap within each tile, with additional space reserved for later vascular layout integration. The proposed grid layout algorithm is a specialised treemap algorithm that utilises the general properties of the hierarchical anatomical system. For our specific use case, we have simplified the overall algorithmic approach by not distinguishing the size or ordering of children of a parent node. This improves the overall readability and fast retrieval of information on the body’s hierarchical anatomical system through the treemap layout.

The algorithm generally operates on a tile-by-tile basis (i.e., separate on each subgraph), with each subgraph associated to a tile laid out independently. First, the root node is identified within the graph of the corresponding tile. Then, a treemap layout, starting at the root node, is carried out in a top-down approach, where the children of each node are allocated progressively smaller space in the corresponding tile. For the layout, two types of grids can be chosen: a dynamic grid that adapts to the children of the node and a fixed global grid for all tiles, with the user deciding which grid to use. With this approach, we aim, similar to the Squarrified algorithm, for an aspect ratio close to one. The result can be seen in Figure 2.

### 4.2 Vascular layout

In the second step of the visualisation pipeline, vascular data for the circulatory paths is integrated, enhancing the treemap with detailed vascular information. A vascular layout refers to the arrangement of a vascular graph representing the blood vessel system information. Vascular layouts are tied to the graph for the treemap layout, which must contain contact points, such as microcirculations, to connect with the vascular layout. Note that the information for the vascular and treemap layouts are distinct graphs and do not share any nodes. Edges in the vascular graph should be orthogonally oriented when possible. These segments are connected at specific points, termed *bend points*, which are calculated during the vascular layout process.

In general, the vascular layout should fit into the treemap representation, focusing on five main goals:

1. *independence* to simplify the overall problem by breaking it into manageable parts;
2. *shortest paths* to ensure direct and efficient routing without unnecessary detours;
3. *anatomical accuracy* to faithfully represent the actual structure of the vascular system, requiring precise positional data;
4. *clarity* to prevent overlapping and maintain clear distinctions between arteries and veins; and
5. *completeness* to fully match the layout of nodes and edges of the original graph, ensuring a thorough and accurate representation.

To enhance clarity in the layout, venous and arterial vessels are separated within each tile. A complete microcirculation is depicted with arterial edges (red, carrying oxygen-rich blood from the heart) and venous edges (blue, draining oxygen-poor blood toward the heart). In the treemap, blue (venous) vessels are always positioned on the right side, while red (arterial) vessels are placed on the left. The edges are routed to the tiles horizontally or vertically, through top or bottom channels, with a colour-coded separation (red at the top; blue at the bottom). This setup includes designated vascular channels above and below the treemap in each tile, ensuring the visibility of the treemap is maintained without interference. Since lower channels are reserved for blue (venous) paths, and upper channels are reserved for red (arterial) segments, L-shaped areas are created around the treemap based on the vessel type.

### 4.3 Vascular layout method

The integration of a vascular layout interconnected with the representation of an anatomical hierarchy can be divided into the four following steps:

1. identifying tile-specific transition points (called hubs);
2. generating internal layouts from the leaves of the vascular graph;
3. calculating positions for nodes outside the vascular graph; and
4. creating the external layout connecting to the heart chambers

It should be noted that the structure of the vascular graph is quite complicated with only a part of the nodes clearly assignable to specific tiles. The layout process has to deal with the layout within the tiles (separated between arterial and venous vessels), the connection of nodes within tiles to nodes outside tiles, and the overall connection of the vascular system to the heart.

#### 4.3.1 Identifying tile-specific transition points

**Figure 6:**
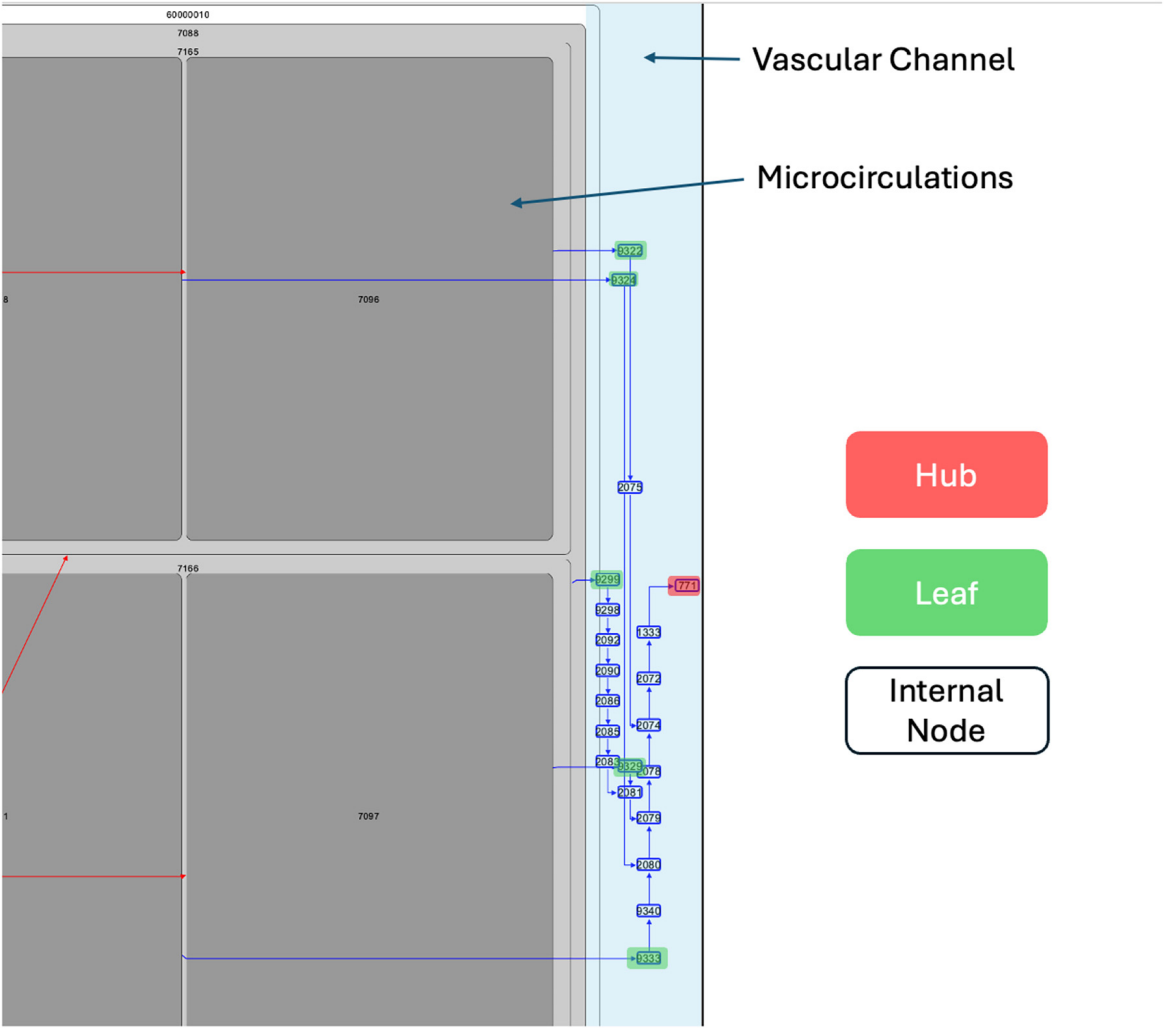
Part of a vascular layout of a tile showing a vascular channel, hubs, and internal nodes.

In the first step, to avoid overlapping paths and guide them through specific areas of the treemap to their target microcirculation, transition points (the so-called hubs, see Figure 6) are identified in the vascular graph. Starting from the leaves of the vascular graph, each leaf (end of a vascular path at a node within the treemap) is assigned a unique cluster ID based on its target microcirculation. The bottom-up approach propagates the cluster ID from the leaves to their parent nodes, identifying hubs (the end-point of this process) as nodes with a single cluster ID passed from their descendants within one tile. Hubs are inside tiles and represent points where paths converge from the leaves to the root, they are prioritised in the layout process. The hubs (see Figure 6) can be seen as starting points for vessels inside the tile. They are crucial in separating the layout process into internal and external tile layouts.

#### 4.3.2 Generating internal layouts from the leaves of the vascular graph

The second step of the vascular layout (internal layout) operates on the given vascular graph, divided into subtrees based on the microcirculation regions within the treemap. The hub of each region, as determined in the previous step, is already part of the existing layout. We also know that a corresponding microcirculation exists in the treemap for each leaf of the given subgraph.

Starting from the leaves of each subtree (representing the endpoints of microcirculation paths), the paths are constructed in a bottom-up approach towards the hub until either reaching the hub itself or a node already laid out. For each path, the horizontal and vertical distances are calculated to compute the layout of the nodes along the path, aiming for a non-overlapping arrangement. The vertical distance between internal nodes is adjusted to ensure even spacing along the path.

#### 4.3.3 Calculating positions for nodes outside the vascular graph

The external layout is computed in the next step of the vascular layout. This layout is not created independently for each tile but collectively for each branch of the vascular graph. Nodes are positioned according to their vessel type and distance from the root (the heart) within the tile. Similar to the tile-internal layout, paths for the external layout are generated bottom-up. The tiles are sorted depending on their distance from the heart. The edges to child nodes are mostly covered by edges originating from the first nodes of the following tile, minimising the number of necessary edges and simplifying the layout.

#### 4.3.4 Creating the external layout connecting to the four heart chambers

As the final step in the external layout, an edge is created for each existing hub to its parent node. Specialised edge routing is required to ensure that edges originating in the hubs and initial path nodes run only through designated channels, preventing overlapping with the rest of the layout or the treemaps.

In the following we will present a use case showing the result of those for steps for a selected part of the FMA with its vascular system.

## 5 Use case

To show the feasibility of the approach and the quality of the layout, a pipeline prototype has been developed as an Add-on in VANTED [33, 34]. Data can be loaded, and the user is offered filter options to refine the data further as described above for specific visualisation needs. Based on the refined parameters, a treemap graph is constructed. The treemap graph is automatically laid out in a standard grid, respecting the tile boundaries defined during the graph construction phase. To load the vascular data, the user can load a file containing vascular paths. The vascular graphs are integrated so that the vascular pathways are visually connected to the anatomical structures displayed in the treemap.

As use cases, we investigated integrating a vascular layout into a broader anatomical context. It involves depicting a part of the anatomical structure of the human body, extracted from the FMA, showcasing some complexity of a vascular system that is currently possible to display using the prototype and our current preprocessed data set. We identified 517 microcirculations with valid paths as contact points for the subsequent vascular layout integration. Some deficiencies in the base data set were found, including the absence of 254 out of 771 microcirculations (32.94 %). This gap underlines the limitations in our retrieved base data, particularly when considering the confinement of the vascular paths in treemap nodes. Consequently, it is crucial to preprocess and evaluate the data retrieved from the FMA to ensure an accurate layout of the anatomical and vascular system.

The vascular layout includes 925 paths, each originating in one of the heart chambers and ending in a particular microcirculation. Every path is marked by a unique identifier (FMA-ID) for the starting point, followed by a sequence of vascular segments tracing the trajectory of the path. By ensuring that only paths with both the start and end point being included in the treemap, we obtained 616 paths that can be laid out, constituting 66.59 % of the overall paths included in the vascular data set. Conversely, the remaining 33.41 % of paths were excluded due to the above-mentioned unavailability of sufficient data quality within the treemap, highlighting a potential area for further data enhancement and integration.

**Figure 7:**
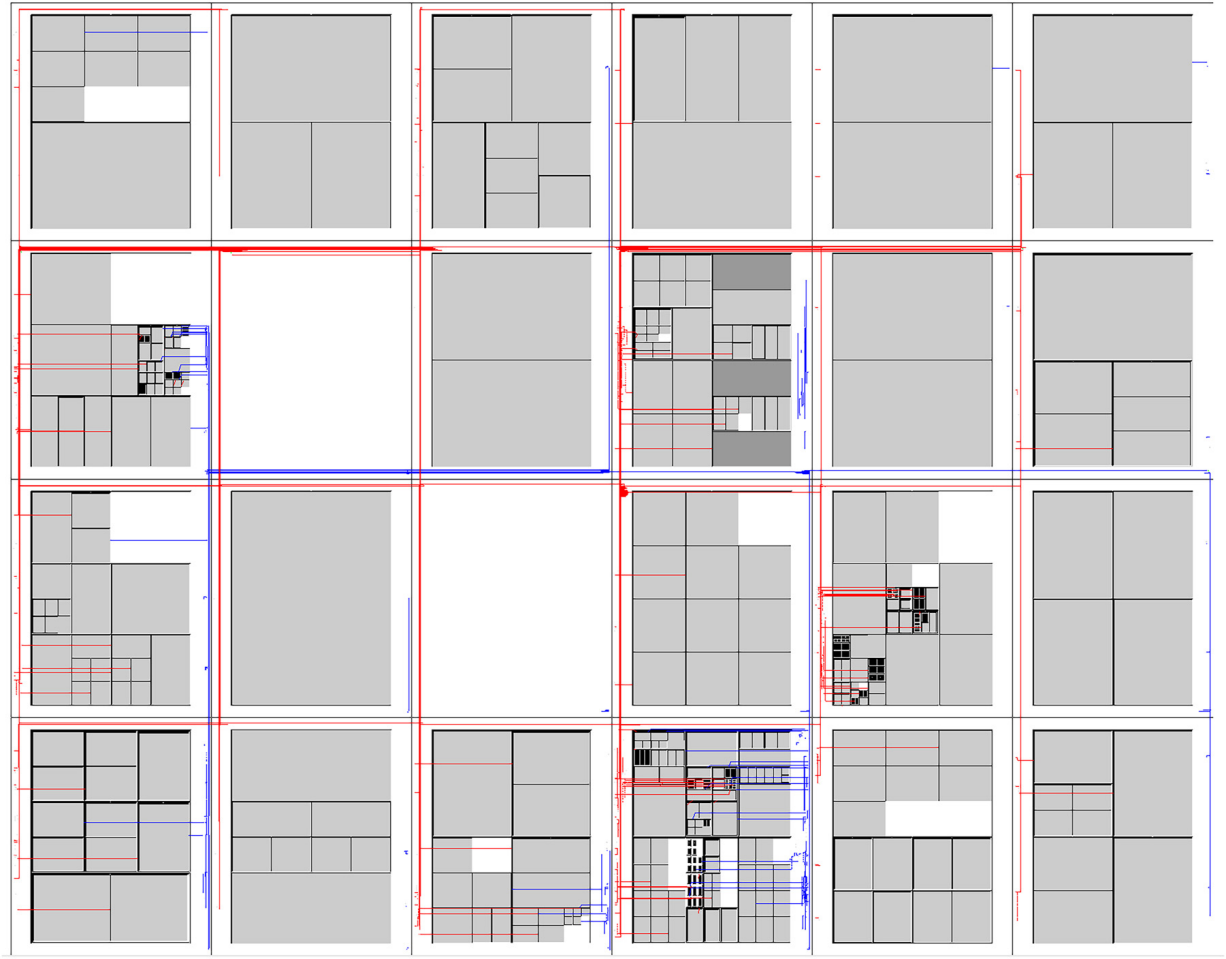
Example of a layout of the FMA ontology including the vascular system for the selected part of the FMA.

Figure 7 shows the layout computed according to our method.

## 6 Discussion and conclusion

This paper showcases a novel approach to the visualisation of the Foundational Model of Anatomy (FMA) ontology also used by the ApiNATOMY tool, focusing on the integration and layout of vascular systems within a hierarchical anatomical framework. The proposed method effectively enhances the clarity and comprehension of complex anatomical and vascular data through the use of treemap layouts combined with layout and edge routing of the vascular graph.

There are several limitations and directions for future research: The current prototype could be extended towards a fully functional web-based application, allowing for wider accessibility and interaction by users across different platforms. Future developments could also incorporate the nervous system into the layout, which also connects elements of the FMA. As anatomical data becomes more detailed, the complexity of the visual representations increases. Developing methods to abstract or simplify these visualisations without losing critical information can be another future development. Techniques such as adaptive detail levels or filtering could be employed to manage this complexity effectively.
